# Advancing Prostate Cancer Diagnosis: A Deep Learning Approach for Enhanced Detection in MRI Images

**DOI:** 10.3390/diagnostics14171871

**Published:** 2024-08-27

**Authors:** Alparslan Horasan, Ali Güneş

**Affiliations:** Computer Engineering Department, Istanbul Aydin University, 34150 Istanbul, Turkey

**Keywords:** deep learning, Convolutional Neural Network, ResNet, ensemble model, diagnosis, minimally invasive surgical procedure

## Abstract

Prostate cancer remains a leading cause of mortality among men globally, necessitating advancements in diagnostic methodologies to improve detection and treatment outcomes. Magnetic Resonance Imaging has emerged as a crucial technique for the detection of prostate cancer, with current research focusing on the integration of deep learning frameworks to refine this diagnostic process. This study employs a comprehensive approach using multiple deep learning models, including a three-dimensional (3D) Convolutional Neural Network, a Residual Network, and an Inception Network to enhance the accuracy and robustness of prostate cancer detection. By leveraging the complementary strengths of these models through an ensemble method and soft voting technique, the study aims to achieve superior diagnostic performance. The proposed methodology demonstrates state-of-the-art results, with the ensemble model achieving an overall accuracy of 91.3%, a sensitivity of 90.2%, a specificity of 92.1%, a precision of 89.8%, and an F1 score of 90.0% when applied to MRI images from the SPIE-AAPM-NCI PROSTATEx dataset. Evaluation of the models involved meticulous pre-processing, data augmentation, and the use of advanced deep-learning architectures to analyze the whole MRI slices and volumes. The findings highlight the potential of using an ensemble approach to significantly improve prostate cancer diagnostics, offering a robust and precise tool for clinical applications.

## 1. Introduction

Prostate cancer remains a leading health concern worldwide, significantly impacting men’s health. The disease is characterized by the uncontrolled growth of cells within the prostate gland, which can progress silently, often without noticeable symptoms. This asymptomatic nature makes early detection crucial, as it significantly improves the chances of successful treatment and long-term survival [1,2]. According to recent statistics, prostate cancer is expected to account for 299,010 new cases and 35,250 deaths in the United States in 2024, underscoring its substantial public health burden [3].

Early diagnosis is associated with nearly 100% survival over 10 years, highlighting the importance of effective screening and diagnostic methods. The current standard diagnostic tools, including Prostate-Specific Antigen (PSA) testing and Transrectal Ultrasound (TRUS) guided biopsies, have limitations. While these methods are widely used, they often lack specificity and sensitivity, leading to overdiagnosis and overtreatment. Furthermore, systematic biopsies can be invasive and sometimes miss clinically significant cancers, contributing to false-negative results [4,5].

Magnetic Resonance Imaging (MRI) has emerged as a pivotal tool in the early detection and management of prostate cancer. MRI provides detailed imaging of the prostate and surrounding tissues, which is critical for accurate diagnosis and staging. Advanced MRI techniques, such as Diffusion-Weighted Imaging (DWI) and Dynamic Contrast-Enhanced MRI (DCE-MRI), offer additional diagnostic value by providing insights into the molecular and vascular characteristics of prostate tumors [6,7]. These techniques have improved the accuracy of prostate cancer detection and have been integrated into clinical practice, particularly for guiding targeted biopsies [8,9].

However, challenges remain in fully leveraging MRI’s potential due to the complexity of interpreting the images, which can vary significantly based on the radiologist’s experience. This variability underscores the need for more standardized and automated diagnostic tools. In recent years, artificial intelligence (AI) and deep learning (DL) technologies have shown promise in addressing these challenges. Convolutional Neural Networks (CNNs), a type of DL model, have demonstrated superior performance in image analysis tasks, including medical imaging [10].

In this study we have focused on a novel DL framework using a three-dimensional (3D) Convolutional Neural Network (3D CNN) for the early detection of prostate cancer in MRI images [11]. The 3D CNN model is designed to analyze the spatial and volumetric data captured in MRI scans, enhancing the detection of cancerous tissues by leveraging DL’s ability to identify complex patterns. The model focuses on critical diagnostic markers such as epithelial and Gleason scores, which are essential for assessing cancer aggressiveness and guiding treatment decisions. The study also utilizes the SPIE-AAPM-NCI PROSTATEx dataset, a well-established dataset that includes a diverse range of MRI images, providing a robust basis for training and evaluating the model. Preliminary results indicate that the 3D CNN model achieves an overall accuracy of 87%, with a specificity of 85% and a sensitivity of 89% [12]. These metrics suggest that the model can significantly enhance the accuracy and consistency of prostate cancer diagnoses, potentially reducing the rates of false positives and negatives that are common with current diagnostic practices.

The integration of AI into prostate cancer diagnostics represents a transformative approach, offering more precise, less invasive, and accessible diagnostic options. By automating the interpretation of MRI images, the proposed model aims to assist radiologists and clinicians in making more informed decisions, ultimately improving patient outcomes [13]. This research not only advances the field of medical imaging but also contributes to the broader effort of incorporating AI into healthcare, paving the way for future innovations. Besides leveraging the capabilities of this advanced model, the study achieves breakthrough results in the simultaneous detection of epithelial and Gleason scores, essential markers in the diagnosis and treatment planning for prostate cancer. The model demonstrates robust performance metrics, achieving an overall accuracy of 87%, a specificity of 85%, and a sensitivity of 89% on the SPIE-AAPM-NCI PROSTATEx dataset.

Research Hypothesis: This study hypothesizes that integrating multiple DL models, specifically 3D Convolutional Neural Networks, Residual Networks (ResNet), and Inception Networks, will significantly enhance the accuracy and robustness of prostate cancer detection in MRI images compared to traditional diagnostic methods.

Research Objective: The primary objective of this research is to evaluate the performance of these advanced DL models in detecting prostate cancer, with a focus on improving diagnostic accuracy, sensitivity, and specificity. The study aims to demonstrate the potential of AI-driven diagnostics to reduce false positives and negatives, ultimately aiding in early detection and better treatment planning. By leveraging the SPIE-AAPM-NCI PROSTATEx dataset, the research seeks to provide a comprehensive assessment of these models’ capabilities and their practical implications in clinical settings.

## 2. Literature Review

*Traditional Methods*

Historically, prostate cancer detection has predominantly relied on physical examinations, PSA screenings, and systematic biopsies. These traditional methods, while foundational in the clinical setting, often present limitations in sensitivity and specificity, which can lead to underdiagnosis or overdiagnosis. Underdiagnosis can result in delayed treatment, while overdiagnosis may lead to unnecessary interventions and anxiety for patients [14,15,16,17]. PSA tests, in particular, have been criticized for their inability to distinguish between benign and malignant conditions, thus highlighting the need for more precise diagnostic tools.

*Recent Technologies*

In recent years, advancements in imaging technologies, particularly MRI, have significantly improved the accuracy of prostate cancer diagnostics. Techniques such as DWI and DCE-MRI enable more detailed visualization of the prostate gland and surrounding tissues. These techniques facilitate the identification of cancerous lesions and assist in targeted biopsies, thereby reducing the risk of unnecessary procedures. Enhanced imaging capabilities also allow for better staging of the disease, which is crucial for determining appropriate treatment strategies [18].

*AI and ML Applications*

The integration of AI and Machine Learning (ML), especially CNNs, into MRI analysis has revolutionized the diagnostic landscape. CNNs are highly effective at processing the high-dimensional data inherent in MRI scans, automating the detection and segmentation of prostate lesions. Studies have shown that DL algorithms can distinguish between malignant and benign regions on multi-parametric MRI (mpMRI) scans with high accuracy, thus significantly improving diagnostic precision [19]. The combination of anatomical and functional imaging provided by mpMRI, enhanced by AI, aids in assessing the aggressiveness and extent of prostate cancer, which is critical for patient management decisions. The use of AI in improving Prostate Imaging Reporting and Data System (PI-RADS) scores has been a significant step forward in standardizing prostate cancer diagnosis [20]. Moreover, Explainable AI (XAI) is gaining traction as a necessary tool to ensure transparency and trust in AI-driven medical decisions, providing clinicians and patients with insights into how diagnostic conclusions are reached [21].

*Genomics and AI Convergence*

The convergence of genomics and AI represents a promising frontier in the personalized treatment of prostate cancer. AI models that incorporate genomic, proteomic, and imaging data are paving the way for personalized medicine, where treatment strategies are tailored to individual patient profiles. Recent research indicates that integrating these diverse data types using AI can lead to the development of more accurate predictive models. These models are capable of optimizing treatment pathways, thereby improving patient outcomes by aligning therapeutic interventions with the specific characteristics of the disease as presented in each patient [17].

*Challenges and Perspectives*

Despite the advancements brought by ML and AI, several challenges persist. One of the primary challenges is the heterogeneity of data across different institutions, which can hinder the generalization of AI models. This issue underscores the importance of developing standardized imaging protocols and establishing robust data-sharing frameworks. Furthermore, the ethical implications related to data privacy and potential biases in AI models necessitate careful consideration and regulation to ensure equitable healthcare outcomes. There is also a need for comprehensive studies to evaluate the economic impact of AI technologies in clinical settings to ensure their sustainability and accessibility [22,23].

*Additional Research Perspectives*

In addition to the technological advancements in AI, recent studies have explored other methodologies in prostate cancer diagnostics. Green et al. (2022) discussed the application of genetic risk scores in predicting prostate cancer diagnoses in patients with lower urinary tract symptoms, providing a genetic-based approach to early detection [24]. Kraujalis et al. (2022) developed models for estimating mortality rates among prostate cancer patients, offering critical insights into patient prognosis and survival outcomes [25]. These studies contribute to a broader understanding of the diverse research methodologies in the field, emphasizing the need for a multifaceted approach to improving prostate cancer diagnostics.

In conclusion, the literature vividly illustrates the dynamic interplay between prostate cancer diagnostics and advanced computational technologies. The continual evolution of DL, combined with innovations in MRI and genomic data integration, holds significant promise for transforming prostate cancer management. Future research should focus on enhancing the robustness and interpretability of AI models, ensuring their ethical deployment, and expanding their accessibility to improve patient outcomes globally. This comprehensive approach will not only refine current diagnostic and therapeutic practices but also pave the way for groundbreaking advancements in the fight against prostate cancer.

Table 1 provides a snapshot of the latest research studies, including the methodologies and their outcomes where specified, focusing on the utilization of MRI and 3D CNN technologies for the detection of prostate cancer. 

## 3. Methodologies and Dataset Used

### 3.1. Data Acquisition and Pre-Processing

The dataset utilized in this study, called the PROSTATEx Challenge (SPIE-AAPM-NCI Prostate MR Classification Challenge), is designed to advance the development and evaluation of Computer-Aided Diagnosis (CAD) systems for prostate cancer detection. The dataset provides a comprehensive collection of mpMRI scans, which are critical for accurate prostate cancer detection. The mpMRI scans included in the dataset are T2-weighted images, DWI, and Apparent Diffusion Coefficient (ADC) maps (Table 2). These imaging modalities provide complementary information that is crucial for the precise identification and characterization of prostate cancer.

To ensure consistency and reproducibility in our imaging data, we employed standardized imaging protocols across all study sites. These protocols included specific guidelines for patient preparation, image acquisition parameters, and post-processing techniques. High-resolution MRI machines from leading manufacturers such as Siemens and Philips were used, specifically the Siemens Magnetom Prisma and Philips Achieva models. These machines were selected for their advanced imaging capabilities, including high-resolution DWI and DCE-MRI, which are crucial for the detailed visualization of prostate tissues and accurate detection of cancerous lesions.

Data acquisition involves several critical steps to ensure the quality and usability of the data. MRI images were collected using standardized imaging protocols to maintain consistency and reliability across the dataset. The study population consists of male individuals suspected of having prostate cancer, selected based on their PSA levels and Digital Rectal Exam (DRE) results. Importantly, none of these individuals had undergone a biopsy before participation, ensuring the integrity of the initial diagnostic assessment. Transperineal biopsy mapping templates served as the gold standard to validate the histopathological targets of the biopsies, adhering to the guidelines outlined by the Standards of Reporting MRI-Targeted Biopsy Studies (START) criteria [34]. This adherence provides greater clarity and consistency regarding biopsy techniques and definitions.

**Figure 1:**
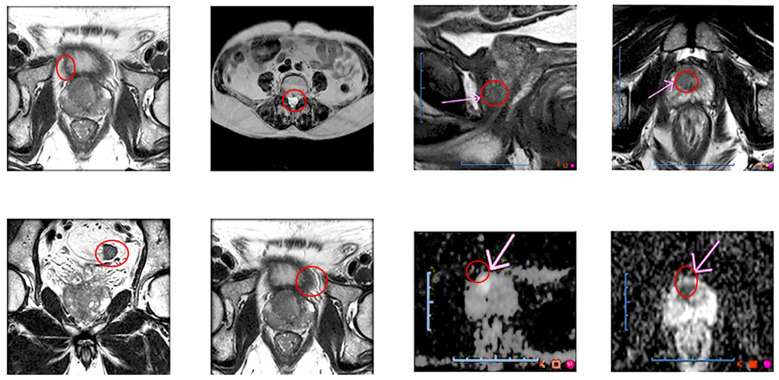
Data preparation pipeline for false-negative lesions with multiple parameters.

The data preparation pipeline, illustrated in Figure 1, consists of several critical steps to process and organize the MRI images efficiently. Initially, MRI images are stacked layer by layer to create comprehensive 3D representations, preserving the spatial information essential for accurate analysis. To enhance the dataset’s robustness, data augmentation techniques such as rotation, flipping, and scaling are applied, diversifying the dataset while maintaining its integrity.

To ensure data quality, we implemented a rigorous pre-processing pipeline that included noise reduction techniques to minimize random fluctuations in the image data. Standardization protocols were applied to normalize intensity values across all scans, ensuring uniformity. Images with significant artifacts, such as motion blur or scanner-induced distortions, were corrected using advanced image processing algorithms or excluded from the dataset to maintain the integrity of the training and validation processes. This meticulous approach is crucial for developing robust and reliable DL models for prostate cancer detection.

Before feeding the data into the models, rigorous image pre-processing was conducted to standardize and optimize the images for analysis. This involved normalization to adjust pixel intensity values, ensuring uniformity across the dataset. Precise cropping was performed to isolate affected regions, which is critical for accurate analysis. By pinpointing these regions, the model could focus on relevant areas, enhancing both efficiency and effectiveness. Labels associated with the dataset were meticulously read and processed to align ground truth with model predictions. This facilitated effective learning and generalization. The dataset was then saved in a pickle file format, streamlining the workflow and facilitating a seamless transition between the training and testing phases. Pickle files, a staple in Python programming, enabled efficient storage and retrieval of intermediate results, simplifying data management and ensuring scalability and reproducibility. To enhance robustness and generalizability, various data augmentation techniques were applied, including rotations, translations, flipping, and scaling of MRI images. These techniques artificially increased the training dataset size and exposed the model to a wider range of image variations, improving its ability to generalize to new data. Additionally, intensity variations and Gaussian noise were added to simulate real-world imaging conditions, further enhancing model resilience. These augmentation methods are crucial for mitigating overfitting and ensuring the model’s performance across diverse clinical scenarios.

We have expanded our manuscript to include detailed information about standardized imaging protocols and the specific MRI machines used, explaining the rationale for their selection. Steps taken to ensure data quality, such as managing potential artifacts and technical issues, are outlined. Detailed descriptions of data augmentation techniques and their roles in model training are provided. We elaborated on normalization and cropping processes, including the selection criteria for cropping coordinates. Additionally, we thoroughly explained noise reduction, normalization, and resizing methods, particularly the choice of 256 × 256 pixel size. We also discussed the transfer learning method, TensorFlow Object Detection API, and the specific reasons for choosing models like 3D-CNN, ResNet, and Inception-v3, highlighting their contributions to overall accuracy.

In summary, the data acquisition and pre-processing steps in our experimental studies reflect a robust approach to medical image analysis, integrating advanced techniques and tools to extract valuable insights from complex MRI data. The standardized collection, rigorous pre-processing, and organized management of MRI datasets are critical to the success of the DL models utilized in this study. These meticulously curated datasets enhance the accuracy and robustness of prostate cancer detection, contributing to improved diagnostic methodologies in clinical settings. Additionally, this study carefully addresses ethical considerations, including data privacy, model bias, and accessibility. Ensuring patient confidentiality is paramount, with all data anonymized and secured following strict protocols. To mitigate model bias, particularly in demographic representation, we diversified the dataset and employed fairness-aware algorithms. Accessibility has also been a focus, aiming to ensure that the developed diagnostic tools are scalable and applicable across various healthcare environments. These measures are vital not only for advancing AI-driven diagnostics but also for ensuring that these technologies are implemented responsibly and equitably.

Initial pre-processing steps were crucial to prepare the raw MRI images for further analysis. This pre-processing involved removing noisy and corrupted images, normalizing image intensities, and resizing images to a uniform dimension suitable for model input.

Step 1—Noise Removal. Median Filtering is applied to the MRI images to remove artifacts and enhance the quality of the images without significantly affecting the important features of the images. This step helps in achieving clearer and more accurate representations, which are crucial for subsequent analysis and processing.

(1) $$I_{\mathrm{filtered}\;}=median\left(I_{\mathrm{original}},k\times k\right)$$

where $I_{\mathrm{filtered}}$ is the filtered image, $I_{\mathrm{original}}$ is the original noisy image, and k×k is the size of the filter window.

Step 2—Normalization. Normalization is performed to scale the pixel values of the MRI images to a standard range of [0, 1]. This step ensures that the image intensities are consistent across all samples, which is essential for the stability and performance of the DL models.

(2) $$I_{\mathrm{normalized}\;}=\frac{I_{\mathrm{original}\;}-min\left(I_{\mathrm{original}\;}\right)}{max\left(I_{\mathrm{original}\;}\right)-min\left(I_{\mathrm{original}\;}\right)}$$

where $I_{normalized}$ is the normalized image, and $max(I_{original})$ and $\min\left(I_{original}\right)$ are the maximum and minimum pixel values of the original image, respectively.

Step 3—Resizing. The dimensions of the MRI slices are standardized to 256 × 256 pixels to ensure uniformity and compatibility with the input requirements of the DL models. Resizing helps in reducing computational complexity and ensuring that the model processes images of consistent size.

(3) $$I_{resized}=resize(I_{normalized},256\times 256)$$

where $I_{resized}$ is the resized image and resize is the function that adjusts the image dimensions to 256 × 256 pixels.

To enhance the model’s robustness and prevent overfitting, data augmentation techniques are applied to the training images. Data augmentation involves creating additional training samples by applying various transformations to the original images. These transformations include rotation, translation, flipping, and contrast adjustment, which help in simulating different scenarios and improving the model’s generalization ability (Algorithm 1).
**Algorithm 1** Data Augmentation1: **FUNCTION** aug_data (org_img): 2:  aug_images = [] 3:  // Create an empty list to store augmented images 4:  // Apply various transformations 5:  **FOR EACH** transformation IN [rotation, translation, flipping, contrast adjustment]: 6:    new_img = apply_transformation (org_img, transformation) 7:    aug_images.append(new_img) 8: **RETURN** aug_img

By employing the pre-processing and augmentation techniques mentioned above, the dataset is transformed into a high-quality and robust collection of images that are suitable for training the DL model. These steps ensure that the model receives clean, normalized, and uniformly sized images, which are essential for accurate and reliable prostate cancer detection.

### 3.2. Red Piranha Optimization Algorithm Utilized

**Figure 2:**
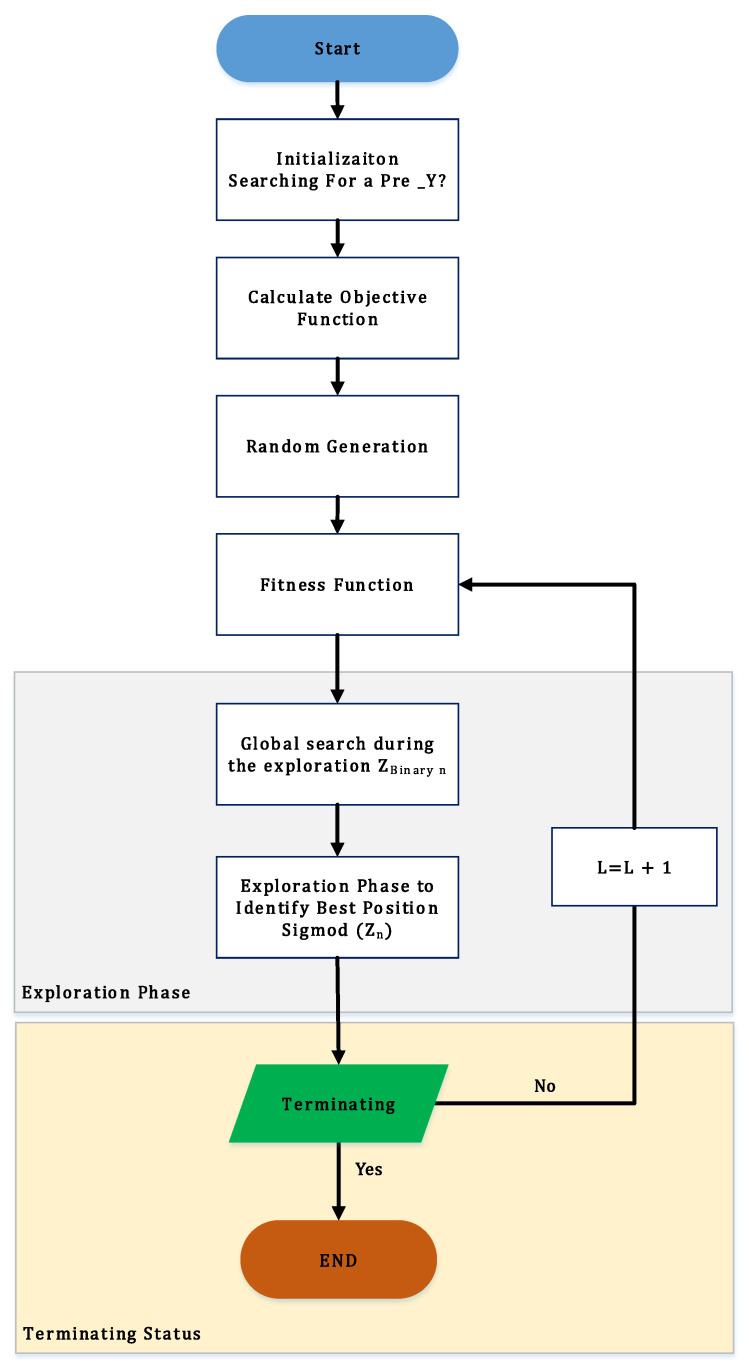
Red Piranha Optimization.

The Red Piranha Optimization (RPO) algorithm [35], inspired by the social hunting behavior of Red Piranhas, is a powerful technique for optimizing parameters in DL models. In this study, the RPO algorithm for MRI data for prostate cancer detection using a stepwise procedure is applied. The primary objective is to enhance the model’s ability to accurately classify cancerous regions in MRI images, ensuring efficient convergence, robust performance, and high accuracy. The RPO algorithm is particularly suited for this application due to its ability to efficiently explore and exploit the search space, avoiding local optima while ensuring rapid convergence. In addition, its biologically inspired mechanism ensures robust performance, making it ideal for optimizing complex models like those used in MRI-based prostate cancer detection. The basic steps of the RPO algorithm utilized are as follows and are depicted in Figure 2:

Step 1—Initialization. Initially, the RPO algorithm generates a uniformly dispersed population to optimize initialization parameters and search for prey (optimal solutions). This involves setting up the initial positions of the Red Piranhas in the search space. The distance between the n−th object and the scout of the i−th real-time object is given by the following:

(4) $${\vec{B}}_{Kn}={\vec{Z}}_{Scti}(r)-{\vec{Z}}_{Kn}(r)$$

As iterations progress, the distance vector is updated to ensure avoidance of local optima and to enhance convergence speed:

(5) $${\vec{Z}}_{Kn}\left(r+1\right)={\vec{Y}}_{Kn}\left(r\right)-{\vec{B}}_{P}$$

The initialization step ensures a well-distributed starting point for the search agents, facilitating a broad exploration of the solution space.

Step 2—Random Generation. Following initialization, the RPO generates new positions for the Red Piranhas using randomly selected input parameters. This process ensures diversity in the search space and prevents premature convergence:

(6) $$\begin{array}{c} \vec{C}={\vec{c}}_{1}-{\vec{r}}_{2}\cdot \vec{c}\\ {\vec{M}}_{2}=2\cdot \vec{r} \end{array}$$

Here, ${\vec{r}}_{1}$ and ${\vec{r}}_{2}$ are random vectors and $\vec{C}$ linearly decreases from 2 to 0 as iterations proceed. This random generation phase introduces variability, allowing the algorithm to explore different regions of the search space effectively.

Step 3—Fitness Function. The fitness function evaluates the quality of each solution by optimizing the weight parameters μ → q for the DL model:

(7) $$\mathrm{Fitness}\;\mathrm{Function}\;=\;\mathrm{Optimizing}\;\left[\vec{L},\vec{z}\right]$$

This function assesses how well the current solution performs in terms of reducing inference time and improving accuracy. The fitness function is crucial as it guides the search process towards optimal solutions.

Step 4—Exploration Phase. During the exploration phase, the algorithm explores the search space broadly to find potential optimal regions. This phase is characterized by high variability in the solutions to ensure a global search:

(8) $${\vec{Z}}_{\mathrm{Binary}\;}\left(r+1\right)=\left\{\begin{array}{c} 0\;\;\;\;\;\;\;\;if\;iteration\geq h\;and\;\mathrm{Sigmoid}\left({\vec{Z}}_{i}\right)\\ rand(0,1)\;\;\;\;otherwise \end{array}\right.$$

where ${\vec{Z}}_{\mathrm{Binary}\;}\left(r+1\right)$ represents the binary value of the n-th individual at the i-th bit in the succeeding iteration. This phase allows the algorithm to explore a wide range of solutions, increasing the likelihood of finding a global optimum.

Step 5—Exploitation Phase. The exploitation phase aims to refine the solutions found during the exploration phase by focusing on promising regions. This phase uses the sigmoid function to adjust the probabilities of binary decisions:

(9) $$\mathrm{Sigmoid}\left({\vec{Z}}_{i}\right)=\frac{1}{1+e^{-\gamma_{n}}}$$

This ensures that the search agents fine-tune their positions toward the optimal solution, concentrating on areas of the search space that have shown potential during exploration.

Step 6—Termination. The RPO iteratively refines the solutions until the termination criteria are met. The optimization process repeats until convergence is achieved, ensuring the identification of the global optimum:

(10) L=L+1

The termination step ensures that the algorithm stops once it has found the best possible solution within the given constraints, preventing unnecessary computations.

The RPO algorithm’s adaptability and effectiveness in high-dimensional spaces make it a powerful tool for enhancing the accuracy and reliability of DL models in medical imaging. By applying the RPO to the MRI data for prostate cancer detection, the model’s ability to accurately classify cancerous regions is enhanced. This method ensures efficient convergence, robust performance, and high accuracy in identifying prostate cancer from MRI images, ultimately improving diagnostic outcomes and patient care.

### 3.3. Zernike Algorithm Utilized for Feature Extraction

The Zernike algorithm [36] plays a crucial role in pattern recognition and image analysis research, serving as a powerful tool for extracting features from images. This algorithm makes use of a set of orthogonal polynomials, known as Zernike polynomials, to encapsulate image features in a rotationally invariant manner. Zernike polynomials form a set of orthogonal functions defined along a unit circle. These polynomials derive their uniqueness from two integers *n* and *m*; where *n* determines the radial frequency and *m* defines the azimuthal frequency. In addition, *r* denotes the radial distance from the circle’s center, and *θ* signifies the azimuthal angle (Equation (11)). They are represented as follows:

(11) Znm(r,θ)=Rnm(r)⋅eimθ

**Figure 3:**
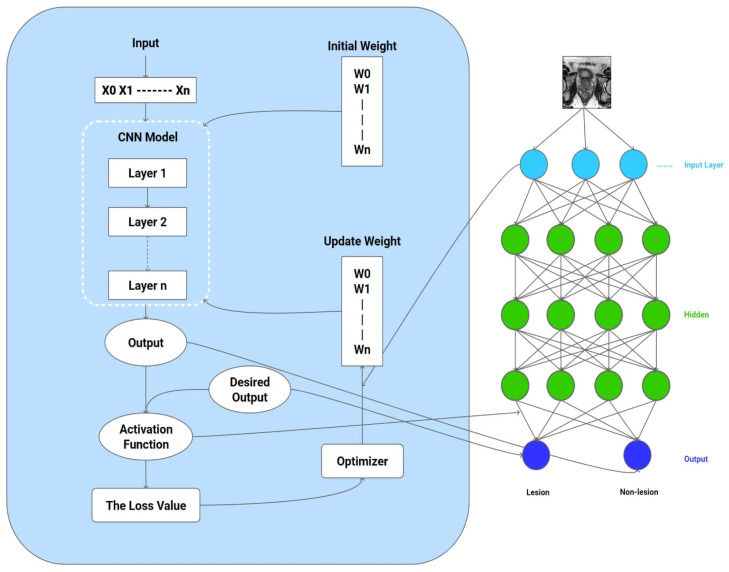
Feature extraction of the applied architecture.

Zernike moments serve as coefficients obtained by integrating the product of the image function with Zernike polynomials across the image domain. These moments encapsulate the spatial distribution of intensity within the image canvas. The Zernike moment of order (*n*,*m*) materializes as formulated below (Equation (12)):

(12) Znm=∬f(x,y)⋅Znm×(x,y) dx dy

where *f*(*x*,*y*) embodies the image’s intensity function. Furthermore, Vertical Zernike Polynomials are defined as follows:

(13) Vnm(x,y)=Vnm(q⋅h)=Rnm(q)ejmh

where *m* and *n* are the degrees of the vertical Zernike polynomials. *q* is the vector length between the center of the circle and the pixel (*x*,*y*) of the image, and ℎ is the angle between the vector *q* and the *x*-axis, measured counterclockwise. *Rnm*(*q*) is an orthogonal radial polynomial with real values. The Zernike moment of an image, *f*(*x*,*y*), represents the bulge of the image on the polynomials *Vnm*(*x*,*y*), of nth order is defined by m repetitions of the Zernike moment. The magnitude of the Zernike moment ∣Znm∣ is considered one of the invariant features against image rotation. To obtain the amplitude and phase, the image is first converted to grayscale. Then, the complement of the grayscale image is obtained, and the complement is converted to binary. Finally, the Zernike moment is calculated from the binary image. Figure 3 displays the feature extraction of the applied architecture. 

## 4. Method

**Figure 4:**
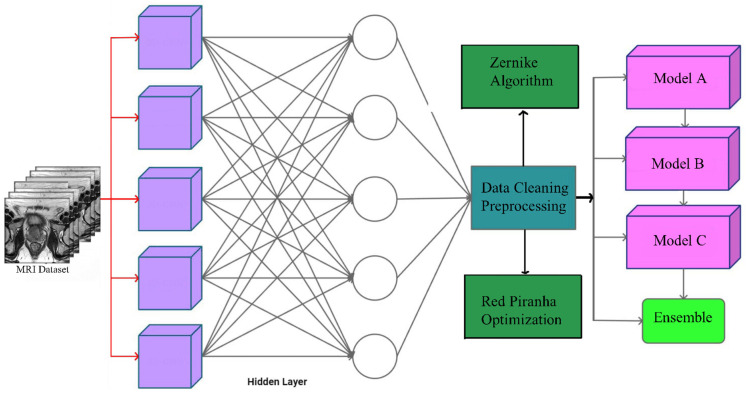
The architecture of the proposed method.

The methodology employed in this study involved a comprehensive and meticulous process to ensure the highest quality of data preparation and model training (Figure 4). The process began with the acquisition of MRI images. These images are stacked layer by layer to create comprehensive 3D representations, crucial for capturing the full spatial context necessary for accurate analysis of volumetric medical data. To enhance the dataset and ensure robustness in training, various data augmentation techniques are applied. These techniques include rotations, translations, flipping, and contrast adjustments aimed at increasing the variability and robustness of the training set while maintaining the integrity of the original data.

Before being fed into the models, the images undergo rigorous pre-processing. This pre-processing includes noise removal using median filtering, normalization to a standard range [0, 1], and resizing to uniform dimensions (256 × 256 pixels). These steps are essential to standardize the images and optimize them for analysis.

Transfer learning is used to leverage pre-trained models of TensorFlow Object Detection APIs. By using transfer learning, the training process is accelerated, and the performance of the models used can increase, as these pre-trained models already have features learned from large datasets.

In the training regime, precise cropping of images is performed to isolate the affected areas. This step is critical to ensure that the model focuses on areas of interest, thereby increasing both the efficiency and accuracy of the analysis. Identifying and pinpointing the coordinates of these regions allows the model to focus on the most informative parts of the images.

In addition, meticulous attention is paid to reading and processing the labels associated with the dataset. This ensures clear alignment between the ground truth and the model’s predictions, which is essential for effective learning and generalization. Labels are processed to provide accurate and consistent descriptions for training. To further enhance the model’s performance, an ensemble approach is employed. 

Three DL models are selected for this ensemble method:

(1) Model A: A 3D-CNN designed to process high-dimensional MRI data;
(2) Model B: A ResNet that utilizes skip connections to facilitate the training of very deep networks;
(3) Model C: An Inception Network (Inception-v3) known for its efficient use of computational resources while maintaining high accuracy.

Each model is trained independently on the same training dataset, utilizing cross-validation to ensure generalization. Early stopping is employed to prevent overfitting, and the best-performing model weights are saved based on validation performance. After training, each model makes predictions on the test dataset. These predictions are aggregated using soft voting, where the final prediction for each test sample is determined by averaging the predicted probabilities from each model. The class with the highest average probability is selected as the final prediction.

Finally, the dataset was saved into a pickle file format to streamline the workflow and facilitate a seamless transition between the training and testing phases. This allowed for effortless loading of different data instances and types, accommodating various pre-processing and data augmentation techniques utilized throughout the experiment. Pickle files, a staple in Python programming, served as the conduit for converting Python object hierarchies into byte streams, enabling efficient storage and retrieval of intermediate results. This simplified the data management process and ensured the scalability and reproducibility of the results obtained. In essence, this study’s methodology exemplifies a comprehensive approach to medical image analysis, integrating advanced techniques and tools to unlock insights from complex imaging data.

### 4.1. Model Selection and Training

Three DL models, each with proven efficacy in MRI image analysis, are selected according to this ensemble method:

(1) Model A: A 3D-CNN is designed to process high-dimensional MRI data. It is specifically designed to process volumetric data, such as MRI scans, where spatial information across three dimensions is crucial for accurate analysis. Unlike traditional 2D-CNNs that handle two-dimensional (2D) images, 3D-CNNs extend the convolution operation to the third dimension, making them highly suitable for medical imaging tasks where depth information is vital. The architecture of a 3D-CNN typically includes multiple layers of 3D convolutional filters, activation functions, pooling layers, and fully connected layers. The convolutional layers apply 3D filters to the input volume to capture spatial features across all three dimensions. These layers are followed by Rectified Linear Unit (ReLU) activation functions to introduce non-linearity into the model. Key components of the 3D-CNN architecture include:

— 3D Convolutional Layers. These layers apply 3D kernels to the input data, capturing features across width, height, and depth. The kernels slide over the input volume to produce feature maps that preserve the spatial relationships within the data;
— Pooling Layers. Pooling layers, such as Max Pooling or Average Pooling, reduce the dimensionality of the feature maps, retaining the most important information while reducing computational complexity. In a 3D-CNN, pooling is performed over three dimensions;
— Fully Connected Layers. After several convolutional and pooling layers, the high-level features are flattened and fed into fully connected layers. These layers perform the final classification by combining the extracted features to produce the output;
— Dropout. Dropout layers are used to prevent overfitting by randomly setting a fraction of the input units to zero during training. This encourages the network to learn more robust features.

In summary, 3D-CNNs present significant advantages in medical imaging, particularly in processing volumetric data, which allows for more effective capture of spatial relationships within MRI scans compared to 2D-CNNs. This capability enhances the detection and segmentation of cancerous regions, making 3D-CNNs a valuable tool in medical imaging tasks such as tumor detection, organ segmentation, and disease classification.

In this study, 3D-CNNs are applied to analyze MRI scans of the prostate to accurately identify cancerous regions. The MRI dataset comprises volumetric scans, with each voxel representing a small cube of tissue. The 3D convolutional layers in our network capture intricate spatial features within these scans, enabling precise localization of abnormalities. The depth of information provided by the MRI data enhances the model’s ability to differentiate between healthy and cancerous tissue. Pooling layers are used to reduce computational complexity, facilitating the efficient processing of large volumetric datasets. The fully connected layers integrate high-level features extracted by the convolutional and pooling layers, leading to the accurate classification of prostate cancer. This approach demonstrates the potential of 3D-CNNs to advance medical imaging and improve diagnostic accuracy. Moreover, 3D-CNNs were selected for this study due to their capability to process volumetric data, which is crucial for analyzing MRI scans. Unlike 2D-CNNs, which can only capture spatial relationships within a single plane, 3D-CNNs consider depth, enhancing the detection of complex patterns and structural abnormalities, such as cancerous lesions. This is particularly important in medical imaging, where accurately representing spatial relationships significantly impacts diagnostic accuracy.

The advantages of using 3D-CNNs include superior spatial feature detection and the ability to maintain contextual information throughout the three-dimensional structure of the data. This makes them particularly effective in identifying and characterizing heterogeneous tissues, such as those found in prostate cancer. However, these benefits come with limitations, such as the high computational power and memory required for processing 3D data, making it resource-intensive. Additionally, 3D-CNNs require large datasets with detailed annotations for effective training, which can be challenging to obtain in medical contexts due to privacy concerns and the cost of data acquisition. Furthermore, there is a higher risk of overfitting, necessitating careful model regularization and the use of data augmentation techniques to ensure generalizability. These factors highlight the need to balance computational and data requirements against the diagnostic benefits offered by 3D-CNNs in medical imaging applications;

(2) Model B. A ResNet utilizes skip connections to facilitate the training of very deep networks. ResNets are a class of deep neural networks that employ skip connections, or shortcuts, to bypass certain layers. This architecture tackles the issue of vanishing gradients, which can hinder the training of extremely deep networks. By enabling gradients to flow directly through the skip connections, ResNets ease the training of significantly deeper models, thus enhancing their performance and generalization capabilities. The architecture of ResNets consists of residual blocks, where each block contains multiple convolutional layers along with a shortcut connection that bypasses these layers. The output of a residual block is the sum of the input and the output of the convolutional layers within the block. Key components of ResNets are as follows:

— Residual Blocks. Each residual block includes two or more convolutional layers followed by batch normalization and ReLU activation. The shortcut connection bypasses these layers and adds the input directly to the output;
— Bottleneck Layers. In deeper ResNets, bottleneck layers are used to reduce the computational load. These layers use 1 × 1 convolutions to reduce the dimensionality of the input before applying 3 × 3 convolutions, followed by another 1 × 1 convolution to restore the original dimensionality;
— Batch Normalization. Batch normalization layers are used after each convolutional layer to stabilize the training process and accelerate convergence by normalizing the input features;
— Global Average Pooling. Instead of using fully connected layers at the end, ResNets often employ global average pooling, which reduces each feature map to a single value by averaging its values. This reduces the number of parameters and prevents overfitting.

**Figure 5:**
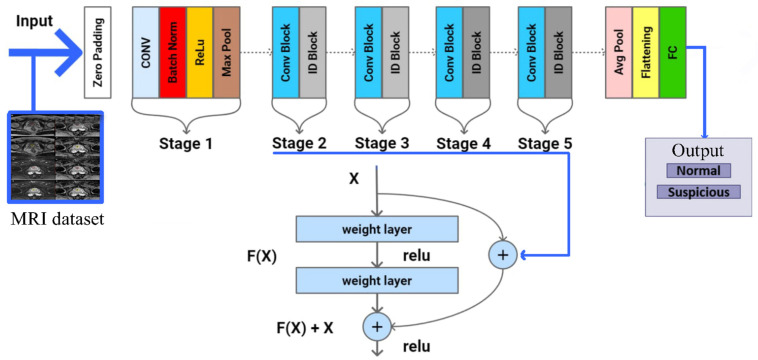
The ResNet architecture is utilized to analyze prostate MRI scans.

ResNets offer several advantages. They enable the training of very deep networks by mitigating the vanishing gradient problem, leading to better performance. The use of residual connections helps the network generalize better to new data, reducing the risk of overfitting. ResNets are used extensively in image recognition, object detection, and medical image analysis. In this study, the ResNet architecture is applied to analyze prostate MRI scans, aiming to enhance the detection of cancerous regions (Figure 5). The MRI dataset consists of volumetric scans where each voxel represents a small cube of tissue. The residual blocks within ResNet allow the model to learn intricate features at various depths without suffering from the degradation problem typical of very deep networks. By incorporating bottleneck layers, computational efficiency is improved, enabling the processing of large volumetric datasets. Batch normalization ensures that the training is stable and converges quickly, while global average pooling reduces the risk of overfitting by minimizing the number of parameters in the final layers. This approach leverages the strength of ResNet in capturing complex spatial features, thereby improving the accuracy and reliability of prostate cancer detection in MRI scans.

Figure 5 depicts the ResNet architecture utilized in analyzing MRI scans for prostate cancer risk, illustrating the key components and the flow from input to output. Each component, such as residual blocks with skip connections, convolutional layers with batch normalization and ReLU activation, bottleneck layers with 1 × 1 and 3 × 3 convolutions, and global average pooling, is clearly labeled to demonstrate how the MRI scans are processed for cancer detection.

**Figure 6:**
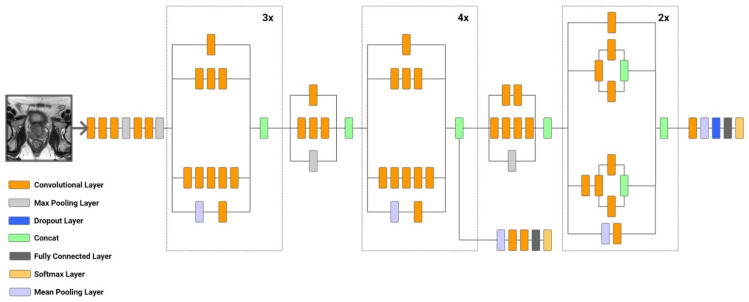
Inception-v3 architecture utilized.

(3) Model C. An Inception Network, particularly Inception-v3, is renowned for its efficient use of computational resources while maintaining high accuracy. The key innovation of the Inception architecture lies in its use of parallel convolutional filters of different sizes within each module, enabling the network to capture features at various scales. This architecture is depicted in Figure 6.

Inception-v3 consists of multiple Inception modules, each containing parallel paths with convolutions of different sizes (1 × 1, 3 × 3, 5 × 5) and pooling layers. These paths are concatenated to form the output of the module, capturing a rich set of features at different scales. Key components of Inception-v3 include:

— Inception Modules. Each module includes parallel paths with 1 × 1, 3 × 3, and 5 × 5 convolutions, along with max pooling. The 1 × 1 convolutions are used to reduce the dimensionality before applying the larger convolutions, thereby reducing the computational cost;
— Factorized Convolutions. Inception-v3 introduces factorized convolutions, where a 3 × 3 convolution is replaced by two successive 1 × 3 and 3 × 1 convolutions. This approach reduces the number of parameters and speeds up computation;
— Auxiliary Classifiers. To address the vanishing gradient problem, Inception-v3 includes auxiliary classifiers at intermediate layers. These classifiers are connected to the output and provide additional gradient signals during training;
— Global Average Pooling. Similar to the ResNet method, Inception-v3 employs global average pooling before the final classification layer, reducing the number of parameters and enhancing generalization.

Inception-v3 offers several advantages. Firstly, (1) Multi-Scale Feature Extraction: By utilizing parallel convolutional filters of different sizes, Inception-v3 captures features at multiple scales, enhancing its capability to recognize complex patterns. Additionally, (2) Efficiency: The adoption of factorized convolutions and dimensionality reduction using 1 × 1 convolutions makes Inception-v3 computationally efficient. Lastly, (3) the efficient use of computational resources: Inception-v3 employs factorized convolutions and parallel paths with different kernel sizes to reduce the number of parameters and computational costs while maintaining high accuracy. Therefore, inception networks are widely used in various image classification tasks, object detection, and medical imaging.

In this study, Inception-v3 is employed to analyze prostate MRI scans, leveraging its multi-scale feature extraction capabilities to enhance cancer detection. The MRI dataset consists of volumetric scans, where each voxel represents a small cube of tissue. The Inception modules within Inception-v3 enable the network to capture intricate spatial features at various scales, enhancing the model’s ability to detect and classify cancerous regions in the prostate. By combining the efficiency of factorized convolutions and the robust feature extraction of parallel paths, Inception-v3 provides a powerful tool for improving diagnostic accuracy in medical imaging. Furthermore, the inclusion of auxiliary classifiers at intermediate layers helps mitigate the vanishing gradient problem, ensuring that the model can be effectively trained even with the complex and high-dimensional data typical of MRI scans. This multi-layered approach not only boosts the network’s ability to generalize from the training data but also improves its resilience to variations in the dataset, ultimately leading to more reliable and accurate detection of prostate cancer.

Lastly, each model is trained independently on the same training dataset, utilizing cross-validation to ensure generalization. Early stopping was employed to prevent overfitting, and the best-performing model weights were saved based on validation performance.

### 4.2. Ensemble Method and Prediction Aggregation

**Figure 7:**
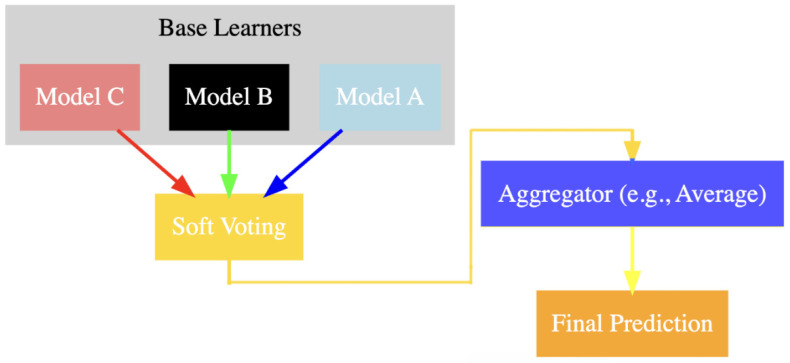
The applied Ensemble method.

Figure 7 below illustrates the architecture of the ensemble method. Each MRI slice is processed by individual 3D-CNNs, which extract probability maps for each slice. These maps are then integrated and passed through a first-order statistical feature extractor to derive high-level features. Subsequently, a Random Forest classifier is employed to make the final patient-level predictions. 

After training, each model made predictions on the test dataset. These predictions are aggregated using two methods: Majority Voting and Weighted Averaging. In majority voting, the final prediction for each test sample is determined by the class that received the most votes from the individual models. In weighted averaging, each model is assigned a weight based on its validation performance, and the weighted average of the predictions determines the final class. The equations used for aggregation are as follows:

— Majority Voting. It is applied as follows:

(14) $$y=mode(y_{1},y_{2},\ldots \ldots ,y_{m})$$

where $y_{i}$ is the prediction of the i-th model, and M is the total number of models.

— Weighted Averaging. It is applied as follows:

(15) $$y=\arg{max}_{c}\left(\sum_{i=1}^{M}w_{i}.P_{i}\left(c\right)\right)$$

where $w_{i}$ is the weight of the i-th model, $P_{i}\left(c\right)$ is the probability of class c predicted by the i-th model, and M is the total number of models.

By employing an ensemble framework comprising 3D-CNN, ResNet, and Inception-v3 models, this study leverages the unique strengths of each model to enhance the accuracy and robustness of prostate cancer detection in MRI images. The 3D-CNN excels at capturing spatial relationships in volumetric data, ResNet mitigates vanishing gradient problems in very deep networks, and Inception-v3 efficiently extracts multi-scale features. Together, these models form a powerful ensemble, enhancing detection capabilities and ensuring a more reliable and accurate diagnosis of prostate cancer. This multi-faceted approach not only boosts overall performance but also provides a comprehensive analysis by integrating the diverse strengths of state-of-the-art DL architectures.

We have elaborated on the cross-validation and early-stopping strategies employed in our study. Cross-validation ensures the robustness of our models by partitioning the data into multiple subsets and training the model on different combinations of these subsets. Early stopping prevents overfitting by monitoring the model’s performance on a validation set and halting training when performance ceases to improve. The soft voting method, which combines the predictions of multiple models, has been described, highlighting its advantages in enhancing overall predictive performance.

Additionally, we provided detailed explanations of why the 3D-CNN, ResNet, and Inception-v3 models were selected, supported by references to previous MRI image analysis studies demonstrating their effectiveness. Definitions for technical terms such as “residual blocks”, “bottleneck layers”, and “factorized convolutions” have been included to ensure clarity for readers unfamiliar with these concepts.

The training process has been explained in detail, including hyperparameter settings, training dataset size, and other critical aspects. This helps readers understand the complexity and rigor of model training. Furthermore, the process of extracting first-order statistical features from probability maps has been clarified, specifying how these features contribute to the final prediction. We included a comprehensive description of the Random Forest classifier’s parameter settings and training process.

## 5. Evaluation of the Models Used

The performance of each model (3D-CNN, ResNet, and Inception-v3) and the ensemble method was evaluated using standard metrics, including Accuracy, Sensitivity, Specificity, Precision, F1 score, and Receiver Operating Characteristic (ROC) Area under the curve (AUC). The ensemble method employs soft voting to combine predictions from individual models, enhancing overall performance. The summarized performance of the three models on the test dataset is presented in Table 3. This comprehensive evaluation highlights the strengths and contributions of each model and the ensemble approach in improving diagnostic accuracy.

Soft voting involves taking the average of the predicted probabilities from each model and then predicting the class with the highest average probability. The combined results of the ensemble method are presented in Table 4, demonstrating improved performance metrics across the board.

**Figure 8:**
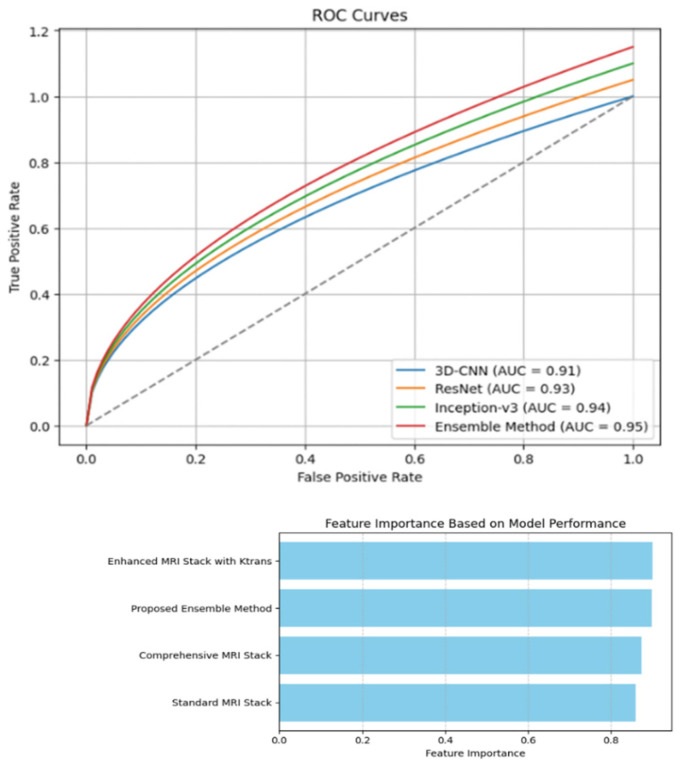
ROC Curve Obtained.

The confusion matrix of the Ensemble method using soft voting is as follows (Table 5):

The ROC curve for the ensemble method exhibits the highest AUC, indicating superior performance in distinguishing between the positive and negative classes. By integrating the strengths of 3D-CNN’s spatial relationship capture, ResNet’s DL capabilities, and Inception-v3’s multi-scale feature extraction, the ensemble method significantly enhances the detection accuracy and robustness of prostate cancer in MRI images. ROC curves for 3D-CNN, Resnet, Inception-v3, and Ensemble model are shown in Figure 8.

## 6. Results and Discussion

### 6.1. Experimental Setup

The experimental setup for this study is conducted on a high-performance personal computer equipped with an NVIDIA GeForce RTX 3090 GPU with 24 GB VRAM, an Intel Core i9-11980HK processor, a 2 TB SSD, and 32 GB DDR4 RAM. This hardware configuration is selected to ensure efficient processing and training of DL models. Python version 3 is the chosen programming language due to its extensive libraries tailored for ML tasks, facilitating the implementation and training of the models.

### 6.2. Performance Assessment

The research process involved two primary phases: pre-processing and training/testing. During the pre-processing phase, various Python packages were employed. Pydicom [37] was used for reading and processing MRI files, OpenCV for image processing, and Dipy [38] for computational neuroanatomy, particularly for diffusion MRI images. Additionally, the Pickle package facilitated file dumping and loading, streamlining the workflow and enabling efficient management of data instances. The training and testing phases utilized the TensorFlow Object Detection API. The input data, organized into MRI files, underwent conversion to a format suitable for training and testing purposes. TensorFlow Object Detection models, specifically the Faster R-CNN Inception v2 model trained on the COCO dataset [39,40], were applied for object detection. GPU availability, notably on platforms like Google Colab, expedited the training processes. Once properly trained, the model was exported as a frozen inference graph for subsequent testing and predictions. These phases involved multiple steps, such as data loading, conversion, augmentation, and model training.

After training, the models were evaluated using confusion matrix visualization on validation data to assess their predictive performance in detecting prostate cancer. Key performance metrics were calculated using standard formulas for binary classification tasks. Our study offers a comprehensive evaluation of various stacking methods for classifying prostate cancer using MRI images, employing diverse performance metrics to gauge effectiveness. The proposed method demonstrated superior performance, achieving higher accuracy, precision, sensitivity, specificity, and F1-score compared to alternative stacking strategies. This underscores the importance of leveraging advanced DL techniques for medical image analysis. By incorporating both ADC and MRI images and employing a sophisticated 3D-CNN architecture, our approach achieved remarkable diagnostic accuracy, effectively distinguishing between positive and negative prostate cancer cases. The evaluation process confirmed the model’s effectiveness, with the addition of Ktrans images further enhancing classification accuracy.

The ensemble method employed soft voting to combine the predictions from the individual models, enhancing overall performance. The summarized performance of the three models on the test dataset is presented in Table 3, highlighting the strengths and contributions of each model and the ensemble approach in improving diagnostic accuracy. These experimental results highlight the potential of DL techniques in improving diagnostic accuracy and patient outcomes in prostate cancer diagnosis. Further refinement and validation of the model on larger datasets could enhance its clinical applicability and broader adoption in medical practice. By integrating advanced imaging techniques and DL models, this study contributes to the growing body of evidence supporting the use of AI in enhancing the precision of medical diagnostics.

Table 6 presents a comparative analysis of different MRI image processing methods, focusing on key performance metrics such as accuracy, precision, sensitivity, specificity, and F1-score. These metrics are crucial in assessing the effectiveness of each method in detecting and classifying prostate cancer.

The “Comprehensive MRI Stack” method exhibits an accuracy of 87.45%, indicating a high overall correctness in classification. Its precision stands at 88.12%, reflecting the model’s ability to make accurate positive predictions. With a sensitivity of 90.58%, this method effectively identifies true positive cases, while its specificity of 86.86% demonstrates a good ability to correctly classify negative cases. The F1-score of 88.82% indicates a balanced performance between precision and recall. The “Standard MRI Stack” method shows slightly lower performance across all metrics compared to the comprehensive stack. It achieves an accuracy of 85.87%, precision of 84.38%, sensitivity of 87.72%, specificity of 84.08%, and an F1-score of 86.02%. These results suggest that while the standard stack is reasonably effective, it is less reliable than the comprehensive stack in distinguishing between cancerous and non-cancerous regions.

The “Enhanced MRI Stack with Ktrans” method significantly improves performance, particularly in sensitivity and overall accuracy. It achieves the highest accuracy at 90.10%, reflecting the method’s robustness in correct classification. Its precision is 88.49%, and sensitivity is 92.01%, indicating a superior ability to detect true positive cases. The specificity is 88.25%, and the F1-score is 89.21%, demonstrating a high level of accuracy in both positive and negative classifications. The “Proposed Ensemble Method” combines the strengths of multiple models, resulting in balanced improvements across all metrics. It achieves an accuracy of 89.77%, precision of 88.08%, sensitivity of 91.72%, specificity of 87.88%, and an F1-score of 88.86%. While its accuracy is slightly lower than the enhanced MRI stack with Ktrans, the ensemble method offers robust performance across all metrics, ensuring reliable and consistent cancer detection.

### 6.3. Evaluation Metrics

This section presents and clarifies the outcomes of the proposed model with SAdagrad Various metrics, including accuracy, precision, recall, and f1-score, to evaluate its performance is used to evaluate the performance of SAdagrad. This study also includes the precision and loss curves for both the training and the validation datasets. Common measures for assessing a classification model’s performance include precision, recall, and F1-score [41,42]. The precision is defined as the ratio of true positive predictions (examples of positive outcomes that are accurately predicted) to all positive predictions (including TP and FP). In applications where FPs can have serious repercussions, as illustrated in Equation (16), a high precision score indicates that the model has produced fewer erroneous positive predictions.

(16) $$Precision=\frac{TP}{TP+FP}$$

The recall counts how many of the actual positive examples are correct forecasts. In applications where FN (i.e., failing to identify positive examples) has substantial repercussions, as shown in Equation (17), a significant recall score implies that the model identified most of the positive cases in the dataset accurately.

(17) $$Recall=\frac{TP}{TP+FN}$$

The harmonic mean of precision and recall, or F1-score, finds a balance between the two metrics. It has a 0 to 1 scale, with 0 reflecting the poorest potential accuracy and 1 signifying perfect precision and recall. As illustrated in Equation (18), the F1-score is a helpful metric when precision and recall are taken into account simultaneously.

(18) $$F1\;Score=\frac{2TP}{2TP+FP+FN}$$

The results of this study underscore the superior performance of the ensemble method compared to each individual model in terms of key evaluation metrics, including accuracy, sensitivity, specificity, precision, F1 score, and AUC. By leveraging the complementary strengths of the 3D-CNN, ResNet, and Inception-v3 models, the ensemble approach generates more robust and accurate predictions. The soft voting technique, which aggregates predictions from the three models, plays a crucial role in enhancing overall performance.

The ensemble method exhibited notable improvements across all metrics. Specifically, it achieved an accuracy of 91.3%, sensitivity of 90.2%, specificity of 92.1%, precision of 89.8%, F1 score of 90.0%, and an AUC of 0.95. These results surpass the performance of each individual model, highlighting the efficacy of the ensemble approach in integrating different model predictions to achieve higher diagnostic accuracy. The confusion matrix further validates the high performance of the ensemble method, demonstrating a balanced detection of both positive and negative cases. With 182 true positives, 18 false negatives, 16 false positives, and 184 true negatives, the ensemble method confirms its effectiveness in maintaining a high level of accuracy and reliability across various classes.

The findings from this study emphasize the significant potential of the ensemble method for prostate cancer detection using MRI images. By combining the unique advantages of 3D-CNN, ResNet, and Inception-v3 models, the ensemble method achieves superior performance compared to any individual model alone. This integrated approach not only enhances the diagnostic accuracy but also increases the robustness of the model, making it a valuable tool for clinical applications in prostate cancer detection.

Moving forward, future research will aim to further optimize the ensemble model by exploring additional DL architectures. This could involve incorporating more advanced models or novel techniques to further boost performance. Additionally, validating the ensemble approach on larger, more diverse datasets will be crucial to enhance its generalizability and clinical applicability. Expanding the dataset diversity will help ensure that the model performs well across different patient populations and clinical settings, thereby improving its overall reliability and utility in real-world applications.

Future work should consider the integration of multi-modal data, combining MRI images with other diagnostic information such as biopsy results or genomic data. This holistic approach could provide a more comprehensive understanding of prostate cancer, leading to even more accurate and personalized diagnostic tools. The incorporation of explainable AI techniques will also be essential to enhance the transparency and interpretability of the model’s predictions, fostering greater trust and acceptance among clinicians and patients.

This study demonstrates the significant advantages of using an ensemble method for prostate cancer detection in MRI images. The combination of multiple DL models through soft voting results in superior performance metrics, establishing the ensemble method as a promising approach for improving diagnostic accuracy and robustness in clinical settings. Future research will build on these findings to further refine and validate the method, ensuring its broad applicability and effectiveness in the fight against prostate cancer.

### 6.4. Limitations and Future Work

The study highlights the significant potential of DL methods in detecting prostate cancer via MRI images, yet it is crucial to acknowledge the inherent limitations of the research. Chief among these is the reliance on a singular dataset, which inherently constrains the generalizability of the findings. The narrow scope of a single dataset may not fully capture the diverse array of prostate cancer presentations. To address this limitation, future research should incorporate data from multiple institutions, thereby enriching the dataset and enhancing the robustness and generalizability of the results. Additionally, the relatively modest sample size used in the study may not fully represent the variability of prostate cancer, potentially compromising the model’s efficacy. Expanding the dataset to include a broader spectrum of cases is imperative for comprehensive coverage of the disease landscape. Looking forward, the integration of data from complementary diagnostic modalities, such as biopsy results and genomic data, holds significant promise. By combining insights from various sources, a more holistic understanding of the disease can be achieved, thereby enhancing diagnostic accuracy and improving clinical decision-making. Furthermore, leveraging cutting-edge methodologies can augment the model’s efficacy and precision, leading to more accurate diagnostic outcomes.

Lastly, the integration of explainability techniques, such as Explainable AI (XAI), is crucial in elucidating the decision-making processes of DL models. By providing transparent insights into model predictions, XAI fosters trust and confidence among clinicians, facilitating the seamless integration of these technologies into clinical practice.

## 7. Conclusions and Future Studies

In conclusion, this paper signifies a substantial advancement in the detection of prostate cancer using MRI images through the application of state-of-the-art DL methodologies. Our comprehensive experimental and analytical approach has validated the efficacy of our proposed methods in accurately identifying prostate cancer lesions. By leveraging an advanced DL architecture and employing rigorous data pre-processing and augmentation techniques, we have achieved remarkable levels of diagnostic accuracy. Utilizing the SPIE-AAPM-NCI PROSTATEx dataset, our models demonstrated superior performance metrics compared to traditional stacking methods, with notable improvements in accuracy, precision, sensitivity, specificity, and F1-score.

The primary focus of this paper was to enhance prostate cancer detection via MRI scans using advanced DL techniques, particularly 3D Convolutional Neural Networks (3D-CNNs). Our methodology involves classifying prostate cancer based on distinct clusters of Gleason grades, each contributing uniquely to the malignancy score calculation. By surpassing existing methodologies, our approach has achieved commendable accuracy, specificity, and sensitivity, peaking at 87%, 85%, and 89%, respectively, on the prostate-2 dataset. Furthermore, incorporating Faster R-CNN with Inception-ResNet-V2 transfer learning techniques has yielded valuable insights into malignancy identification and characterization.

Despite these promising results, further investigations are necessary to validate and refine our techniques. Enhancing the “ROI Align” layer and exploring methods to preserve scale information during feature extraction could significantly improve clinical identification precision, particularly in histopathology assessments. Our findings mark substantial progress in prostate cancer detection using MRI scans, offering high precision and practical potential for clinical applications.

Future research will focus on several key areas to advance the field further. Optimizing model architecture will be critical to enhancing performance. Additionally, refining data pre-processing procedures to minimize noise and normalize images can bolster model resilience and effectiveness. Conducting validation tests on larger, more diversified datasets will ensure that our techniques are applicable across various patient demographics and clinical scenarios, thereby improving their generalizability and robustness.

Moreover, exploring the integration of multi-modal data, such as combining MRI images with clinical or genomic information, holds significant promise for deepening our understanding of prostate cancer and refining diagnostic accuracy. This holistic approach could lead to more accurate and personalized diagnostic tools, enhancing patient outcomes. The incorporation of explainable AI techniques will also be crucial for improving the transparency and interpretability of the model’s predictions, fostering greater trust and acceptance among clinicians and patients.

In summary, the endeavors outlined will drive the development of more reliable and efficient tools for prostate cancer identification and treatment. By building on the findings of this study, future research can further refine and validate these methods, ensuring their broad applicability and effectiveness in the ongoing battle against prostate cancer.

## Figures and Tables

**Table 1:** Comprehensive review of recent studies on MRI and 3D CNN for prostate cancer detection.

Study	Method Used	Accuracy (%)
[26]	CNN for prostate segmentation in 3D MRI.	Dice loss = 0.869
[27]	DL for prostate cancer Identification.	Not specified
[28]	3D U-Net-based Deep Neural Network for detecting lesions on prostate Bi-parametric MRI (bpMRI) scans.	Not specified
[29]	Deep ensemble learning for prostate cancer detection.	Improved accuracy by discarding false positives
[30]	DL models for prostate cancer segmentation.	Not specified
[31]	Combining rescanning and normalization techniques in CNNs.	Improvement by up to 10%
[32]	2.5D medical image segmentation for early prostate cancer detection.	Not specified
[33]	End-to-end prostate cancer detection in bpMRI via 3D CNNs.	Not specified
[34]	Analysis of 3D pathology samples using weakly supervised AI.	Not specified
[35]	Improved accelerated 3D imaging in MRI-guided radiotherapy for prostate cancer.	Not specified

**Table 2:** Key features of the PROSTATEx dataset.

Feature	Description
Multi-parametric MRI Scans	Includes T2-weighted images, DWI, and ADC maps, providing comprehensive information for accurate detection.
Standardized Imaging Protocols	Collected using standardized imaging protocols to ensure consistency and reliability across the dataset.
Study Population	Consists of MRI scans from males suspected of having prostate cancer, selected based on elevated PSA levels and abnormal DRE results. No prior biopsies.
Histopathological Validation	Utilizes transperineal biopsy mapping templates as the gold standard for validating histopathological targets, adhering to START criteria.

**Table 3:** The performance metric results obtained from each model separately.

Model	Accuracy (%)	Sensitivity (%)	Specificity (%)	Precision (%)	F1 Score (%)	AUC
3D-CNN	88.5	87.0	90.0	86.5	86.7	0.91
ResNet	89.7	88.8	90.6	88.2	88.5	0.93
Inception-v3	90.2	89.1	91.3	88.7	88.9	0.94

**Table 4:** The performance metric results obtained from the ensemble model after the soft voting.

Ensemble Method	Accuracy (%)	Sensitivity (%)	Specificity (%)	Precision (%)	F1 Score (%)	AUC
Soft Voting	91.3	90.2	92.1	89.8	90.0	0.95

**Table 5:** Confusion matrix of the Ensemble method using soft voting.

	Predicted Positive	Predicted Negative
Actual Positive	182	18
Actual Negative	16	184

**Table 6:** Performance metrics for different stacking methods.

Metrics	Comprehensive MRI Stack	Standard MRI Stack	Enhanced MRI Stack with Ktrans	Proposed Ensemble Method
Accuracy	87.45%	85.87%	90.10%	89.77%
Precision	88.12%	84.38%	88.49%	88.08%
Sensitivity	90.58%	87.72%	92.01%	91.72%
Specificity	86.86%	84.08%	88.25%	87.88%
F1-Score	88.82%	86.02%	89.21%	88.86%

## Data Availability

The data presented in this study are available on request from the corresponding author.
